# Association between prior HBV vaccine responsiveness and booster-induced anti-SARS-CoV-2 spike antibody titers following the third BNT162b2 dose in Japanese healthcare workers: A prospective observational study

**DOI:** 10.1097/MD.0000000000051417

**Published:** 2026-11-06

**Authors:** Miwa Misawa, Momoko Iwamoto, Tomoyuki Yamada, Fumiko Kawanishi, Masami Nishihara, Akira Asai, Yusuke Kusaka, Taku Ogawa, Takashi Nakano, Akira Ukimura, Tomio Suzuki

**Affiliations:** a Department of General Medicine, Osaka Medical and Pharmaceutical University Hospital, Takatsuki, Osaka, Japan; b Department of Obstetrics and Gynecology, Kochi Health Sciences Center, Kochi, Japan; c Department of Pharmacy, Osaka Medical and Pharmaceutical University Hospital, Takatsuki, Osaka, Japan; d Department of Nursing, Osaka Medical and Pharmaceutical University Hospital, Takatsuki, Osaka, Japan; e Central Clinical Laboratory, Osaka Medical and Pharmaceutical University, Takatsuki, Osaka, Japan; f Department of Anesthesiology, Osaka Medical and Pharmaceutical University, Takatsuki, Osaka, Japan; g Infection Control Center, Osaka Medical and Pharmaceutical University Hospital, Takatsuki, Osaka, Japan; h Department of Microbiology and Infection Control, Faculty of Medicine, Osaka Medical and Pharmaceutical University, Takatsuki, Osaka, Japan; i Osaka Medical and Pharmaceutical University, Takatsuki, Osaka, Japan; j Hirakata City Hospital, Hirakata, Osaka, Japan.

**Keywords:** antibody response, BNT162b2, booster vaccine, COVID-19 vaccines, healthcare workers, hepatitis B virus vaccine

## Abstract

Inter-individual variability in vaccine-induced humoral responses has been reported even among generally healthy individuals. In our previous study, healthcare workers with poor hepatitis B virus (HBV) vaccine responsiveness were more likely to show a low anti-SARS-CoV-2 spike antibody response after the first BNT162b2 dose. This study examined whether such differences persisted after a third BNT162b2 dose. This was an extended follow-up study of a previously reported prospective observational cohort of Japanese healthcare workers. The primary outcome was the quantitative anti-SARS-CoV-2 spike antibody titer measured 12 weeks after the third BNT162b2 dose. Participants were classified as normal HBV vaccine responders, weak responders, or non-responders. Comparisons across HBV response groups were performed using the Kruskal–Wallis test and multivariable linear regression adjusted for age and sex. A low antibody-response analysis (<15 U/mL) after the first dose was evaluated as a secondary analysis. Among 741 participants with antibody measurements 12 weeks after the third dose, 486 were normal HBV vaccine responders, 229 were weak responders, and 26 were non-responders. The median anti-SARS-CoV-2 spike antibody titer was 891,900 U/mL. Titers did not differ significantly across HBV response groups in the unadjusted analysis (*P* = .3718) or after adjustment for age and sex (global *P* = .3971). Compared with normal responders, the adjusted geometric mean ratio was 0.94 (95% CI, 0.83–1.07) for weak responders and 0.83 (95% CI, 0.60–1.15) for non-responders. By contrast, after the first dose, HBV vaccine non-responders had an increased risk of a low antibody response (<15 U/mL) compared with normal responders (adjusted RR, 1.85; 95% CI, 1.10–3.13). Differences in early anti-SARS-CoV-2 spike antibody responses associated with prior HBV vaccine responsiveness were no longer statistically apparent 12 weeks after the third BNT162b2 dose. These findings suggest convergence of measured humoral antibody responses after booster vaccination, although they do not establish that the underlying biological mechanisms of vaccine hyporesponsiveness were eliminated.

## 1. Introduction

The global spread of severe acute respiratory syndrome coronavirus 2 (SARS-CoV-2), first identified in late 2019, triggered an unprecedented global health crisis. The BNT162b2 mRNA vaccine demonstrated high clinical efficacy and strong antibody responses in clinical trials and real-world studies.^[1,2]^ Nevertheless, substantial inter-individual variability in vaccine-induced humoral responses has been reported. Reduced immunogenicity is particularly well described in patients receiving hemodialysis, solid-organ transplant recipients, and other immunocompromised populations.^[3,4]^ Therefore, identifying host characteristics associated with vaccine responsiveness may help interpret postvaccination immune responses and guide booster strategies.

The hepatitis B virus (HBV) vaccine is among the best-characterized vaccines with respect to variability in immunogenicity. In healthy adults, the standard three-dose series induces protective anti-HBs antibody levels in most recipients, whereas older age, diabetes, chronic kidney disease, immunosuppression, and host genetic factors have been associated with lower responsiveness.^[5–7]^ A minor proportion of otherwise healthy adults also comprises weak responders or non-responders despite vaccination.

Only a few studies have examined whether responsiveness to one vaccine is associated with responsiveness to a different vaccine platform. In our previous study of the same prospective cohort of Japanese healthcare workers at Osaka Medical and Pharmaceutical University Hospital, HBV vaccine non-responders were at increased risk of a low anti-SARS-CoV-2 spike antibody response after the first BNT162b2 dose, although all participants with available measurements after the second dose reached the prespecified antibody-response threshold.^[8]^ These findings suggest that prior HBV vaccine responsiveness reflects inter-individual variation in early humoral responses to mRNA vaccination. However, whether these early differences persist after a third (booster) dose of BNT162b2 remains unclear. This question is relevant because booster vaccination substantially increases antibody levels, while antigenically divergent SARS-CoV-2 variants may require higher neutralizing antibody levels than ancestral strains.^[9,10]^

The present study represents an extended follow-up of the same prospective cohort of Japanese healthcare workers previously reported by Iwamoto et al.^[8]^ We extended follow-up to 12 weeks after the third BNT162b2 dose to determine whether differences in anti-SARS-CoV-2 spike antibody responses according to prior HBV vaccine responsiveness persisted after booster vaccination. The primary outcome was the quantitative anti-spike antibody titer 12 weeks after the third dose, compared across normal HBV vaccine responders, weak responders, and non-responders.

## 2. Methods

### 2.1. Study population and design

This study represents an extended follow-up of a previously reported prospective observational cohort of Japanese healthcare workers who received the BNT162b2 mRNA COVID-19 vaccine. The original study evaluated anti-SARS-CoV-2 spike antibody responses through the second vaccine dose; in the present study, follow-up was extended to 12 weeks after the third BNT162b2 dose.

Healthcare workers at Osaka Medical and Pharmaceutical University Hospital (Osaka, Japan) who were scheduled to receive BNT162b2 were consecutively recruited between March 9 and March 24, 2021, as previously described.^[8]^ Vaccination priority was given to frontline healthcare workers, including nurses, physicians, pharmacists, and technicians, as well as administrative staff with frequent patient contact. Of 1051 healthcare workers recruited, 1032 provided written informed consent.

HBV vaccination history and anti-HBs antibody titers were obtained from employee health records maintained by the university’s occupational health program. Baseline serum samples and self-administered questionnaires were collected immediately before the first BNT162b2 dose. Follow-up serum samples were collected immediately before the second vaccination (3 weeks after the first dose), 4 weeks after the second vaccination, and 12 weeks after the third vaccination. Participant demographic data included age, sex, job category, smoking status, alcohol use, and comorbidities.

Participants were included in each time-point analysis according to the availability of anti-HBs data and the corresponding serum antibody measurements. The primary third-dose analysis included the 741 participants with available anti-SARS-CoV-2 spike antibody measurements at 12 weeks after the third dose. Baseline anti-N antibody positivity was used as an indicator of previous SARS-CoV-2 infection. Participants with baseline anti-N positivity were retained in descriptive analyses and the primary third-dose analysis. For adjusted first-dose analyses and the first-dose low antibody-response analysis, participants with baseline anti-N positivity were excluded. A sensitivity analysis of the primary third-dose outcome was additionally performed after excluding participants with baseline anti-N positivity.

No formal sample size calculation was performed because this was an extended follow-up study of the original cohort; all eligible participants with available measurements at the relevant follow-up time points were included. The longitudinal participant flow and study protocol are summarized in Figures 1 and 2, respectively.

**Figure 1.**
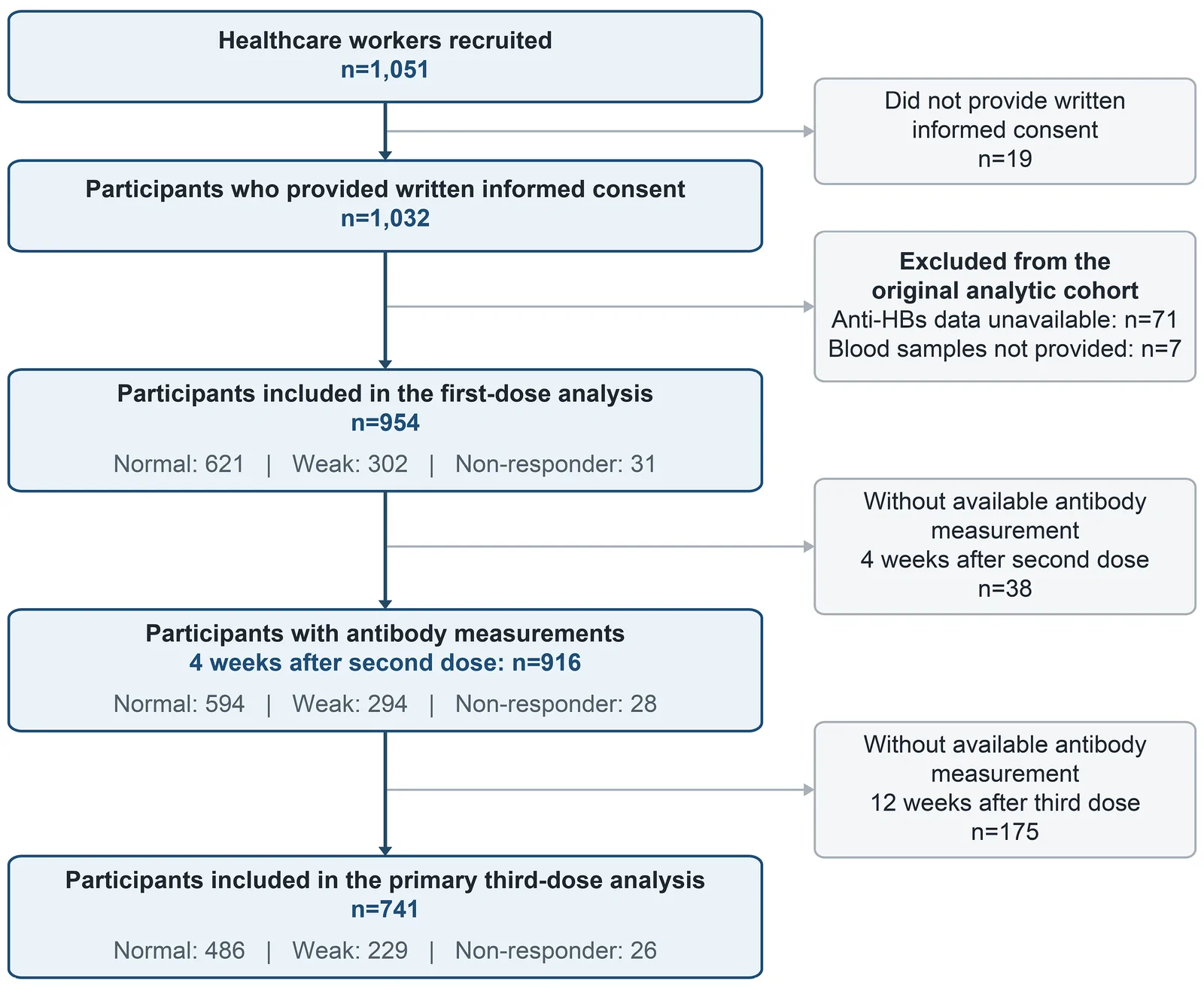
Participant flowchart. Among 1032 consenting healthcare workers, 954 were included in the first-dose analysis, 916 had antibody measurements 4 weeks after the second dose, and 741 were included in the primary 12-week post-third-dose analysis. HBV vaccine-response groups were defined by anti-HBs titers. anti-HBs, antibody to hepatitis B surface antigen; HBV, hepatitis B virus.

**Figure 2.**
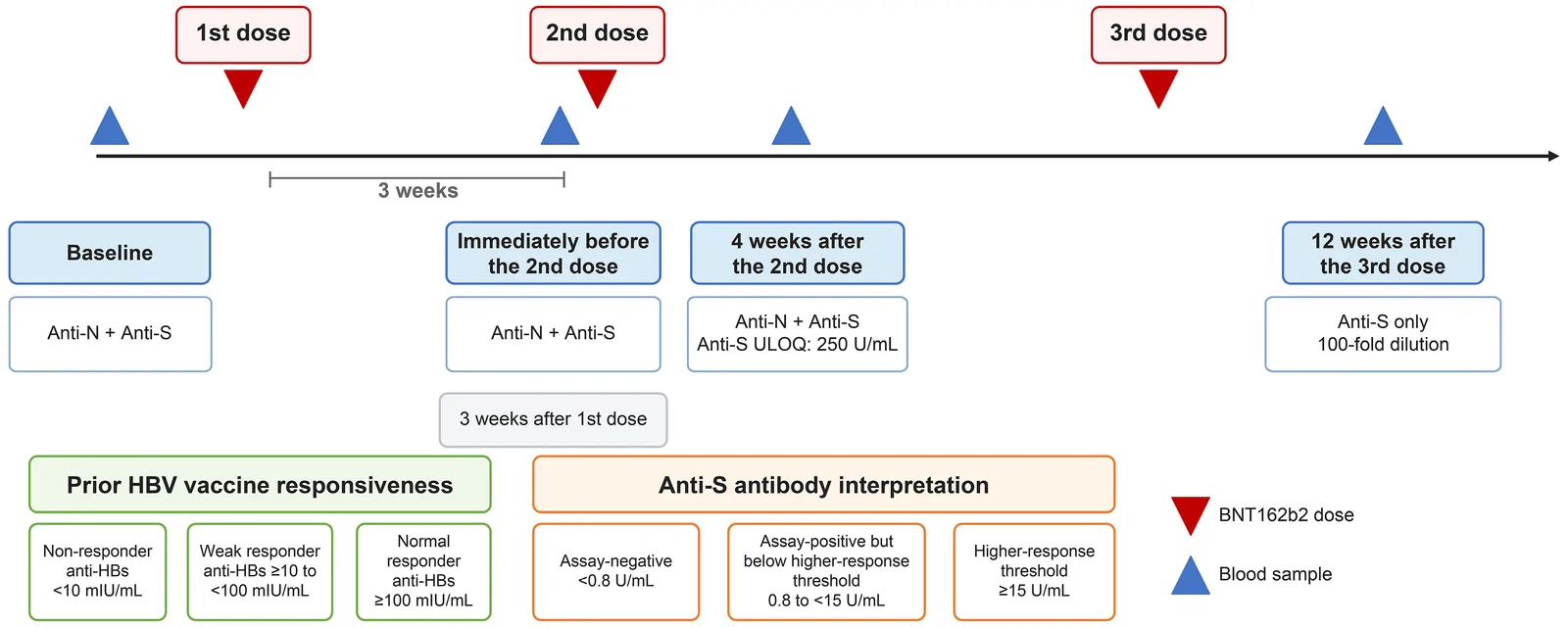
Study protocol for evaluating anti-SARS-CoV-2 antibody responses according to prior HBV vaccine responsiveness. Participants received three doses of BNT162b2. Serum was collected at baseline, immediately before the second dose (3 weeks after the first dose), 4 weeks after the second dose, and 12 weeks after the third dose. antiS antibodies were classified as assay-negative (<0.8 U/mL), assay-positive but below the prespecified higher-response threshold (0.8 to < 15 U/mL), or at/above the higher-response threshold (≥15 U/mL). Anti-N antibodies were used to identify serologic evidence of prior SARS-CoV-2 infection and were not measured 12 weeks after the third dose. At 4 weeks after the second dose, the assay upper limit of quantification was 250 U/mL. Samples obtained 12 weeks after the third dose were measured after 100-fold dilution. HBV vaccine response was classified by anti-HBs titers as nonresponse (<10 mIU/mL), weak response (≥10 to < 100 mIU/mL), or normal response (≥100 mIU/mL).

### 2.2. Antibody testing

Serum samples were analyzed using the Elecsys Anti-SARS-CoV-2 assay, which qualitatively detects antibodies against the SARS-CoV-2 nucleocapsid (N) protein, and the Elecsys Anti-SARS-CoV-2 S assay, which measures antibodies against the receptor-binding domain of the SARS-CoV-2 spike (S) protein (Roche Diagnostics International Ltd., Rotkreuz, Switzerland). Both assays were performed using the Cobas e801 platform at the central laboratory of Osaka Medical and Pharmaceutical University Hospital.

For the anti-N assay, results were considered reactive when the cutoff index (COI) was ≥ 1.0 and non when the COI was < 1.0. For the antiS assay, values ≥ 0.8 U/mL were considered assay-positive according to the manufacturer. An additional prespecified threshold of ≥ 15 U/mL was retained as an operational higher antibody-response threshold, consistent with our previous cohort analysis.^[8]^ In the original cohort, this threshold had been selected based on comparison of findings of the antiS assay with those of a surrogate neutralization assay; in the present extended follow-up, it was retained for consistency and was not treated as a definitive correlate of clinical protection. Prior studies have shown an association between anti-spike assay values and neutralization activity, although exact thresholds vary according to assay and viral variant.^[11]^ Accordingly, categorical analyses distinguished < 0.8, 0.8 to < 15, and ≥ 15 U/mL.

At the time of antibody measurement 4 weeks after the second BNT162b2 dose, the upper limit of quantification of the Elecsys Anti-SARS-CoV-2 S assay was 250 U/mL. Values exceeding this limit were reported as ≥ 250 U/mL and were not further quantified. Therefore, quantitative comparisons of antibody titers above 250 U/mL could not be performed at this time point. For samples collected 12 weeks after the third BNT162b2 dose, antiS antibody measurements were performed after 100-fold dilution.

### 2.3. HBV vaccine response classification

HBV vaccine non-responders were defined as participants with anti-HBs antibody titers < 10 mIU/mL after completion of an HBV vaccination series. Weak responders were defined as those with anti-HBs titers ≥ 10 and < 100 mIU/mL, and normal responders as those with titers ≥ 100 mIU/mL. Participants whose anti-HBs titers reached ≥ 10 mIU/mL only after a second HBV vaccination series were classified as weak responders, consistent with the classification used in the original cohort study.^[8]^ All participants had completed a three-dose HBV vaccination series before December 2020.

### 2.4. Statistical analysis

Continuous variables are presented as median with interquartile range (IQR), and categorical variables as number and percentage. Comparisons across the three HBV vaccine response groups were performed using the Kruskal–Wallis test for continuous variables and the chi-square test or Fisher exact test, as appropriate, for categorical variables.

Because anti-SARS-CoV-2 spike antibody titers were right-skewed, titers were log10-transformed for regression analyses. To evaluate the association between prior HBV vaccine responsiveness and quantitative anti-spike antibody levels, multivariable linear regression models were fitted with log10-transformed antibody titers as the dependent variable and HBV vaccine response category, age, and sex as independent variables. Normal HBV vaccine responders formed the reference group. Robust standard errors were used. Regression coefficients for HBV vaccine response categories were exponentiated as 10^β and are reported as geometric mean ratio (GMR) with 95% confidence interval (CI).

The primary outcome was the quantitative anti-SARS-CoV-2 spike antibody titer measured 12 weeks after the third BNT162b2 dose. The primary comparison was performed across the three predefined HBV vaccine response groups: normal responders, weak responders, and non-responders. The primary adjusted model included age and sex as covariates; no additional covariates were included. All 741 participants with available antibody measurements 12 weeks after the third dose were included in the primary analysis. A sensitivity analysis was performed after excluding participants with baseline anti-N antibody positivity.

For the first-dose analysis, the unadjusted comparison across HBV vaccine response groups was performed in the original cohort of 954 participants. For adjusted analyses after the first dose, participants with baseline anti-N antibody positivity were excluded. In addition to analysis of quantitative antibody titers, the risk of a low antibody response, defined as anti-spike antibody level < 15 U/mL, was evaluated using log-binomial regression adjusted for age and sex. Adjusted relative risk (aRR) with 95% CI is reported.

To assess potential selection bias, age and sex were compared between consenting participants included in the primary third-dose analysis (n = 741) and consenting participants not included in that analysis (n = 291), using the Mann–Whitney *U* test for age and the chi-square test for sex. The prespecified ≥ 15 U/mL higher antibody-response threshold and antibody responses at earlier vaccination time points were considered secondary or supportive measures. Stratified analyses according to age group and HBV vaccine response category were performed using the Kruskal–Wallis test. All statistical tests were two-sided, and results with a *P* value of < .05 were considered statistically significant. The statistical analyses were performed using Stata version 15.1 (StataCorp LLC, College Station, TX), and figures were created using R version 4.1.0 (R Foundation for Statistical Computing, Vienna, Austria).

### 2.5. Ethical review and consent

Before participant enrollment, emergency authorization to conduct this urgent COVID-19-related study was granted by the President of Osaka Medical and Pharmaceutical University under a special institutional procedure. Formal review and approval by the Ethics Committee of Osaka Medical and Pharmaceutical University were subsequently completed (Institutional Review Board No. 2020-163). All participants provided written informed consent before participation.

## 3. Results

A total of 1051 healthcare workers were recruited, of whom 1032 provided written informed consent. Seventy-one participants were excluded because anti-HBs antibody data were unavailable, and seven did not provide blood samples, leaving 954 participants in the original cohort. Antibody measurements were available for 916 participants at 4 weeks after the second BNT162b2 dose and for 741 participants at 12 weeks after the third dose. The latter 741 participants comprised the primary analytic cohort for the third-dose analysis (Fig. 1).

To examine potential selection bias, we compared the data of the 741 consenting participants included in the primary third-dose analysis with those of the 291 consenting participants who were not included. Median age was similar between included and excluded participants (35 [IQR, 28–47] vs 35 [IQR, 30–45] years; *P* = .206). However, the included participants comprised a higher proportion of female individuals than the excluded participants (63.4 vs 37.5%; *P* < .001).

Among the 741 participants included in the primary third-dose analysis, the median age was 35 (IQR, 28–47) years, and 470 (63.4%) were female individuals. A total of 486 participants were classified as normal HBV vaccine responders, 229 as weak responders, and 26 as non-responders (Table 1). Ten participants (1.3%) were anti-N antibody positive at baseline.

**Table 1:** Participant characteristics and antibody responses to severe acute respiratory syndrome coronavirus 2 before and after BNT162b2 vaccination among healthcare workers included in the 12-week post-third-dose analysis (n = 741).

Characteristic/ time point	Total (n = 741)	Normal HBV vaccine response (n = 486)	Weak HBV vaccine response (n = 229)	HBV vaccine nonresponse (n = 26)	Global *P* value	Test
Age, median (IQR), years	35 (28–47)	36 (28–46)	33 (26–46)	51 (43–56)	<.001	Kruskal–Wallis
Female sex, n (%)	470 (63.4)	317 (65.2)	135 (59.0)	18 (69.2)	.219	Chi-square

Nucleocapsid-protein antibody positivity, n (%)						
Baseline	10 (1.3)	8 (1.6)	2 (0.9)	0 (0.0)	.660	Fisher exact
3 weeks after the first dose	8 (1.1)	6 (1.2)	2 (0.9)	0 (0.0)	1.000	Fisher exact
4 weeks after the second dose	8 (1.1)	6 (1.2)	2 (0.9)	0 (0.0)	1.000	Fisher exact
12 weeks after the third dose	Not measured	—	—	—	N/A	N/A

Spike-protein antibody assay positivity (≥0.8 U/mL), n (%)						
Baseline	10 (1.3)	8 (1.6)	2 (0.9)	0 (0.0)	.660	Fisher exact
3 weeks after the first dose	738 (99.6)	485 (99.8)	228 (99.6)	25 (96.2)	.086	Fisher exact
4 weeks after the second dose	741 (100.0)	486 (100.0)	229 (100.0)	26 (100.0)	N/A	N/A
12 weeks after the third dose	741 (100.0)	486 (100.0)	229 (100.0)	26 (100.0)	N/A	N/A

Higher spike-protein antibody response (≥15 U/mL), n (%)						
Baseline	5 (0.7)	4 (0.8)	1 (0.4)	0 (0.0)	1.000	Fisher exact
3 weeks after the first dose	628 (84.8)	420 (86.4)	191 (83.4)	17 (65.4)	.017	Fisher exact
4 weeks after the second dose	741 (100.0)	486 (100.0)	229 (100.0)	26 (100.0)	N/A	N/A
12 weeks after the third dose	741 (100.0)	486 (100.0)	229 (100.0)	26 (100.0)	N/A	N/A

Spike-protein antibody quantitative response, median (IQR), U/mL						
Baseline	0.0746 (0.0726–0.0772)	0.0749 (0.0726–0.0773)	0.0740 (0.0724–0.0759)	0.0762 (0.0737–0.0795)	.004	Kruskal–Wallis
3 weeks after the first dose	53.1 (26.2–131)	56.25 (28.2–128)	50.6 (22.7–138)	38.0 (9.72–90.6)	.087	Kruskal–Wallis
4 weeks after the second dose	250 (250–250)†	250 (250–250)†	250 (250–250)†	250 (250–250)†	N/A	N/A
12 weeks after the third dose	891,900 (530,700–1422,800)	895,550 (532,800–1479,700)	854,800 (544,500–1325,200)	790,400 (423,500–1172,400)	.372	Kruskal–Wallis

Values are presented as median (IQR) or n (%). All data in this table are restricted to the 741 participants included in the primary 12-week post-third-dose analysis; analyses of the original first-dose cohort (n = 954) are reported separately in the text and Figure 3. An anti-spike antibody level ≥ 0.8 U/mL indicates assay positivity according to the manufacturer. The prespecified ≥ 15 U/mL threshold was used as an operational higher antibody-response threshold and should not be interpreted as a definitive correlate of clinical protection.

*P* values represent global comparisons across the three HBV vaccine response groups and were calculated using the Kruskal–Wallis test for continuous variables and the chi-square test or Fisher exact test, as appropriate, for categorical variables. No pairwise *P* values are presented in this table.

Nucleocapsid-protein antibodies were not measured at 12 weeks after the third dose.

HBV = hepatitis B virus, IQR = interquartile range, N/A = not applicable.

† At 4 weeks after the second BNT162b2 dose, the assay upper limit of quantification was 250 U/mL. Values exceeding this limit were censored at 250 U/mL in the analytic dataset; therefore, the displayed medians and interquartile ranges are affected by a marked ceiling effect and no quantitative between-group *P* value is reported.

After the first BNT162b2 dose, anti-SARS-CoV-2 spike antibody titers differed significantly across the predefined age groups (Kruskal–Wallis *P* = .0001; Fig. 3A). Across the three HBV vaccine response groups, antibody titers also differed significantly in the unadjusted analysis (Kruskal–Wallis *P* = .0263; Fig. 3B). However, among participants who were anti-N negative at baseline (n = 943), the overall association between HBV vaccine response category and log10-transformed antibody titers was no longer statistically significant after adjustment for age and sex (global *P* = .1149). Compared with normal responders, the adjusted GMR was 0.91 (95% CI, 0.78–1.07; *P* = .247) for weak responders and 0.57 (95% CI, 0.31–1.05; *P* = .069) for non-responders.

**Figure 3.**
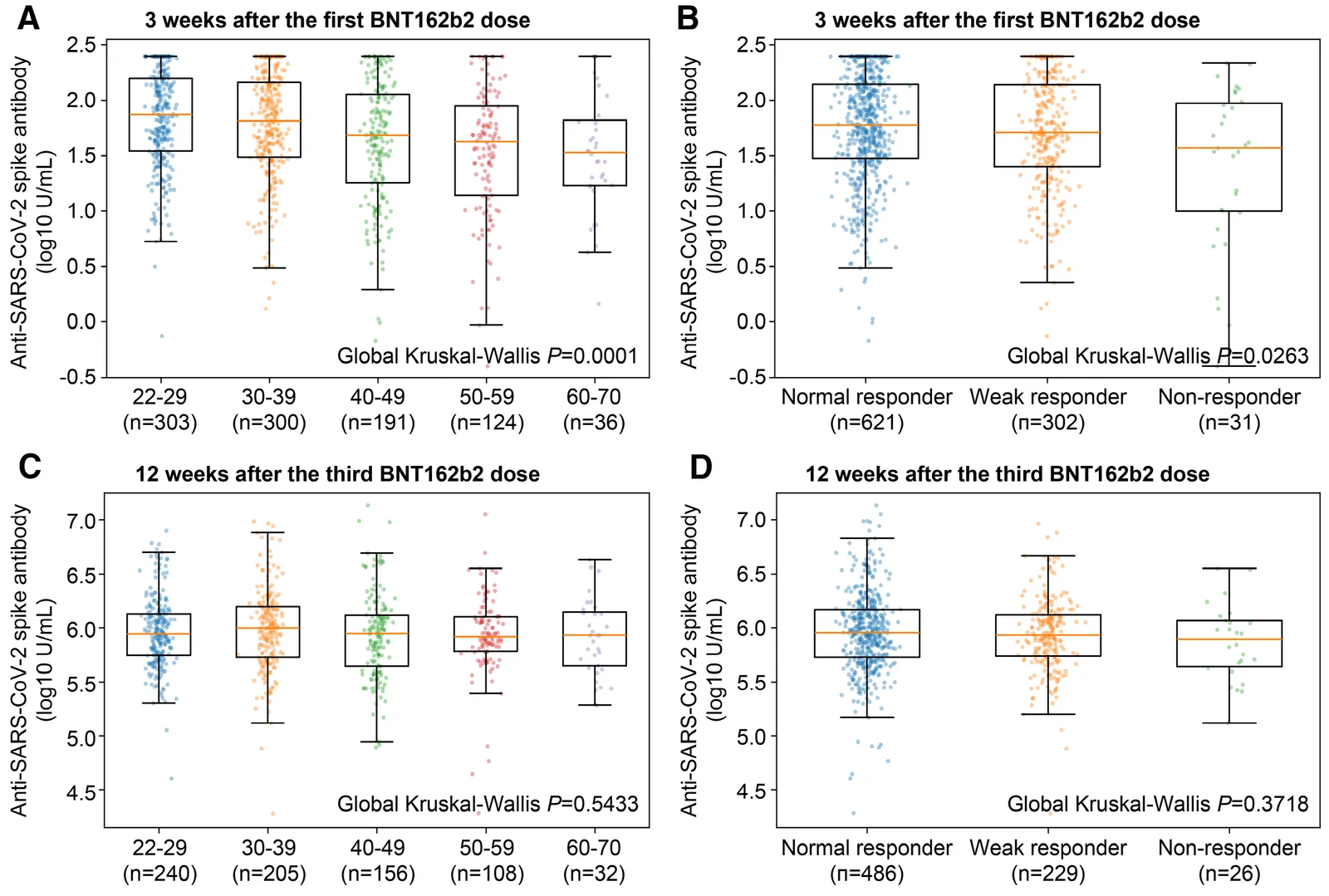
Anti-SARS-CoV-2 spike antibody titers after the first and third BNT162b2 doses, stratified by age and prior hepatitis B virus vaccine response. (A) Antibody titers 3 weeks after the first dose by age group (n = 954: 22–29 years, n = 303; 30–39 years, n = 300; 40–49 years, n = 191; 50–59 years, n = 124; 60–70 years, n = 36). (B) Antibody titers 3 weeks after the first dose by HBV vaccine response (normal responder, n = 621; weak responder, n = 302; non-responder, n = 31). (C) Antibody titers 12 weeks after the third dose by age group (n = 741: 22–29 years, n = 240; 30–39 years, n = 205; 40–49 years, n = 156; 50–59 years, n = 108; 60–70 years, n = 32). (D) Antibody titers 12 weeks after the third dose by HBV vaccine response (normal responder, n = 486; weak responder, n = 229; non-responder, n = 26). Dots represent individual participants; boxes indicate the interquartile range with the median line, and whiskers extend to 1.5 times the interquartile range. *P* values are from global Kruskal–Wallis tests (A, *P* = .0001; B, *P* = .0263; C, *P* = .5433; D, *P* = .3718); no pairwise comparisons were performed. HBV vaccine response was classified by anti-HBs titers as normal (≥100 mIU/mL), weak (≥10 to < 100 mIU/mL), or nonresponse (<10 mIU/mL). HBV, hepatitis B virus; anti-HBs, antibody to hepatitis B surface antigen.

In the complementary analysis of a low antibody response (<15 U/mL) after the first dose among baseline anti-N-negative participants (n = 943), HBV vaccine non-responders had a significantly higher risk than normal responders after adjustment for age and sex (adjusted relative risk [aRR], 1.85; 95% CI, 1.10–3.13; *P* = .021), whereas weak responders did not (aRR, 1.17; 95% CI, 0.84–1.63; *P* = .345).

At 4 weeks after the second BNT162b2 dose, all 916 participants had antiS antibody levels ≥ 15 U/mL. Because 99.3% of participants had values above the assay upper limit of quantification of 250 U/mL, quantitative between-group comparisons at this time point could not be reliably performed. This ceiling effect precluded assessment of differences in antibody magnitude across HBV vaccine response groups after the second dose.

At 12 weeks after the third BNT162b2 dose, the median anti-SARS-CoV-2 spike antibody titer was 891,900 (IQR, 530,700–1422,800) U/mL. The antibody titers did not differ significantly across the predefined age groups (22–29, 30–39, 40–49, 50–59, and 60–70 years; Kruskal–Wallis *P* = .5433; Fig. 3C) or across the three HBV vaccine response groups (*P* = .3718; Fig. 3D). All 741 participants had antiS antibody levels ≥ 15 U/mL.

In the primary multivariable analysis adjusted for age and sex, HBV vaccine response category was not significantly associated with log10-transformed anti-spike antibody titers 12 weeks after the third dose (global *P* = .3971). Compared with normal responders, the adjusted GMR was 0.94 (95% CI, 0.83–1.07; *P* = .357) for weak responders and 0.83 (95% CI, 0.60–1.15; *P* = .262) for non-responders. A sensitivity analysis excluding the 10 participants who were anti-N antibody positive at baseline yielded similar results (n = 731; global *P* = .3651). No new anti-N antibody positivity was observed during the available follow-up measurements through 4 weeks after the second dose.

## 4. Discussion

In this extended follow-up study of a previously reported cohort of Japanese healthcare workers, anti-SARS-CoV-2 spike antibody responses associated with prior HBV vaccine responsiveness differed after the first BNT162b2 dose but were no longer statistically distinguishable 12 weeks after the third dose. After the first dose, the unadjusted quantitative antibody titers differed across HBV vaccine response groups; however, this overall association was attenuated after adjustment for age and sex. In the complementary categorical analysis, HBV vaccine non-responders nevertheless remained at increased risk of a low antibody response (<15 U/mL) compared with normal responders after adjustment for age and sex. In contrast, after the third dose, neither the unadjusted nor the age- and sex-adjusted analyses demonstrated a statistically significant association between prior HBV vaccine responsiveness and quantitative anti-spike antibody titers.

These findings extend our previous report of the same cohort.^[8]^ In the original study, HBV vaccine non-responders were more likely to have a low antibody response after the first BNT162b2 dose than normal responders, suggesting that responsiveness to a previous vaccine may reflect inter-individual variation in early humoral responses to a newly encountered antigen. The present analysis indicates that this association was no longer statistically apparent 12 weeks after the third BNT162b2 dose. The adjusted GMRs after the third dose were 0.94 for weak responders and 0.83 for non-responders compared with normal responders, with confidence intervals including unity. Thus, the measured circulating antibody responses appeared to converge after repeated antigen exposure.

This convergence should be interpreted cautiously. The absence of a significant difference after the third dose does not establish that the biological mechanisms underlying HBV vaccine hyporesponsiveness were eliminated. In particular, the number of HBV vaccine non-responders was small, and the confidence interval around the effect estimate in this group remained relatively wide. Consequently, modest or clinically relevant differences cannot be completely excluded. Our findings therefore support convergence of the measured humoral response at the evaluated time point rather than biological equivalence among the HBV vaccine response groups.

Age was an important potential confounder because HBV vaccine hyporesponsiveness is associated with increasing age and is also known to influence COVID-19 vaccine immunogenicity. Systematic review evidence indicates age, sex, serostatus, and underlying comorbidities as contributors to variation in humoral responses after BNT162b2 vaccination.^[12]^ In the present study, adjustment for age and sex attenuated the association between HBV vaccine response group and quantitative antibody titers after the first dose. At 12 weeks after the third dose, antibody titers did not differ significantly across predefined age groups, and age was not significantly associated with antibody titers in the adjusted model. Residual confounding by unmeasured host factors nevertheless remains possible.

Interpretation of the antibody response after the second dose is limited by the assay characteristics at that time point. Four weeks after the second dose, all participants reached the prespecified ≥ 15 U/mL antibody-response threshold, and 99.3% had values above the assay upper quantification limit of 250 U/mL. The resulting ceiling effect prevented reliable quantitative comparison among HBV vaccine response groups. Therefore, the present data cannot determine precisely when the group differences observed after the first dose diminished between the second and third vaccinations.

The high antibody levels observed after the third dose are consistent with evidence that booster vaccination can markedly increase humoral responses, including against antigenically divergent variants.^[9,10]^ At the same time, circulating antibody levels decline over time after mRNA vaccination, as summarized in longitudinal systematic review data.^[13]^ The ≥ 15-U/mL threshold used in the present study should not be interpreted as a definitive correlate of clinical protection. Rather, it was retained as a prespecified operational threshold for consistency with our previous analysis. The principal outcome of the present study was the quantitative antibody titer after the third dose rather than attainment of this threshold.

Some limitations should be considered. First, this was a single-center study involving Japanese healthcare workers, which may limit generalizability. Second, the study assessed humoral immunity using circulating anti-spike antibody titers and did not evaluate SARS-CoV-2–specific T-cell responses or memory B-cell responses. Therefore, convergence of serum antibody titers across HBV vaccine response groups should not be interpreted as evidence that cellular immunity or immunologic memory also converged; differences in these immune compartments may persist even when circulating antibody levels become similar. Third, attrition occurred during longitudinal follow-up, and selection bias cannot be excluded. Although age was similar between participants included and those excluded from the primary third-dose analysis, the sex distribution differed substantially. Fourth, the relatively small number of HBV vaccine non-responders reduced precision for this subgroup. Fifth, quantitative comparison after the second dose was limited by the assay ceiling effect. Finally, although age and sex were included in the primary adjusted analyses, residual confounding by other host characteristics, including comorbidities and behavioral factors, remains possible.

Despite these limitations, this study has several strengths. It represents prospective longitudinal follow-up of a well-characterized healthcare worker cohort with documented HBV vaccination history and anti-HBs antibody responses, enabling direct comparison of vaccine responsiveness across different antigen exposures. In addition, both unadjusted and adjusted analyses, together with a sensitivity analysis excluding participants with baseline anti-N positivity, support the robustness of the primary findings. Future studies incorporating cellular immunity, memory B-cell responses, and longer-term antibody kinetics are needed to determine whether the observed convergence extends to broader and more durable immune protection.

In conclusion, differences in anti-SARS-CoV-2 spike antibody responses associated with prior HBV vaccine responsiveness were evident after the first BNT162b2 dose but were not statistically apparent 12 weeks after the third dose. These findings suggest convergence of measured humoral antibody responses following booster vaccination, even among individuals with a history of weak or no HBV vaccine responsiveness, but do not establish elimination of the underlying biological mechanisms responsible for vaccine hyporesponsiveness.

## Acknowledgments

The authors would like to express their gratitude to the Medical Statistics Laboratory at Osaka University of Medical and Pharmaceutical Sciences for their support and advice.

## Author contributions

**Conceptualization:** Miwa Misawa, Momoko Iwamoto, Akira Ukimura.

**Data curation:** Miwa Misawa, Momoko Iwamoto, Tomoyuki Yamada, Fumiko Kawanishi, Masami Nishihara, Akira Asai, Yusuke Kusaka, Takashi Nakano.

**Formal analysis:** Miwa Misawa, Momoko Iwamoto, Taku Ogawa.

**Investigation:** Miwa Misawa, Momoko Iwamoto, Tomoyuki Yamada, Fumiko Kawanishi, Masami Nishihara, Akira Asai, Yusuke Kusaka, Takashi Nakano.

**Methodology:** Miwa Misawa, Momoko Iwamoto, Taku Ogawa.

**Software:** Miwa Misawa, Momoko Iwamoto.

**Validation:** Miwa Misawa, Momoko Iwamoto.

**Visualization:** Miwa Misawa, Momoko Iwamoto.

**Writing – original draft:** Miwa Misawa.

**Writing – review & editing:** Miwa Misawa, Momoko Iwamoto, Taku Ogawa, Akira Ukimura.

**Supervision:** Akira Ukimura, Tomio Suzuki.
